# Examining the face validity of the EQ-HWB-9 in dementia: caregiver interpretation across “Today” and “7-Day” recall periods

**DOI:** 10.1186/s12955-026-02478-z

**Published:** 2026-01-28

**Authors:** Maresa Buchholz, Lidia Engel, Feng Xie, Bernhard Michalowsky

**Affiliations:** 1 Patient-Reported Outcomes & Health Economics Research, German Center for Neurodegenerative Diseases (DZNE), Site Rostock/ Greifswald, Ellernholzstraße 1-2, D-17487 Greifswald, Germany; 2 Health Economics Group, School of Public Health and Preventive Medicine, Monash University, Melbourne, 553 St Kilda Rd, Melbourne, VIC 3004 Australia; 3 Department of Health Research Methods, Evidence and Impact, McMaster University, Hamilton, ON Canada; 4 Centre for Health Economics and Policy Analysis, McMaster University, Hamilton, ON Canada

**Keywords:** Dementia, EQ-HWB-9, Health fluctuations, Proxy-proxy perspective, Informal caregivers

## Abstract

**Background:**

This study aimed to assess the face validity of the EQ Health and Wellbeing 9 (EQ-HWB-9) for use as a proxy by informal caregivers of community-dwelling people living with dementia (PlwD). It explored how caregivers understand and interpret the EQ-HWB-9 items when using two different recall periods (today vs. 7-days).

**Methods:**

Qualitative interviews were conducted with 73 informal caregivers of PlwD in Germany. Participants were randomly assigned to one of two recall period groups of the EQ-HWB-9 (“today” vs. “7 days”), and underwent an interview, designed to evaluate the appropriateness of the EQ-HWB-9. Interviews were audio-recorded, transcribed verbatim, and analyzed using qualitative content analysis.

**Results:**

Caregivers were, on average, 70.6 years old and mainly female (72.6%). Most caregivers found the EQ-HWB-9 acceptable, highlighting its broad health coverage, clear structure, and suitability of the five-point response scale. Criticisms included the brevity and insufficient depth to capture the PlwD’s health status fully. Caregivers tended to focus on observable aspects, such as daily activities, concentration, and mobility, while internal states, like loneliness, sadness, and anxiety, were more challenging to assess, often relying on indirect cues. The 7-day recall period was associated with lower adherence and more frequent deviations from recall instructions compared to the “Today” recall. Overall, caregivers adhered well to the instructed proxy-proxy perspective but reported uncertainty in selecting the most appropriate responses.

**Conclusions:**

The EQ-HWB-9 is generally acceptable and feasible for proxy assessments in PlwD. However, challenges regarding recall periods, proxy interpretation, and response certainty require further investigation.

**Supplementary Information:**

The online version contains supplementary material available at 10.1186/s12955-026-02478-z.

## Background

Dementia remains one of the most significant societal and public health challenges facing healthcare systems worldwide. The number of people affected is projected to rise, reaching an estimated 152 million globally by 2050 [1]. In the absence of a cure, maintaining or improving the quality of life for people living with dementia (PlwD) is a central goal in providing appropriate care throughout the disease journey. Quality of life is inherently subjective; the individuals themselves should ideally assess it [2]. However, in health conditions characterized by cognitive decline, such as dementia, self-reports become increasingly unreliable or impossible as the disease progresses. In such cases, proxy assessments – ratings provided by another person – are commonly used [3]. For individuals with dementia, proxies are most often informal caregivers, typically family members who take on the responsibility of care. A substantial body of literature has examined proxy assessments, highlighting strengths and limitations across various generic and disease-specific instruments among different health conditions [4–7].

The EQ Health and Wellbeing (EQ-HWB) is a 25-item generic instrument recently developed to measure a wide spectrum of health and well-being outcomes relevant to economic evaluations across healthcare, public health, and social care settings [8]. The measure intends to reflect aspects of health and wellbeing related to health conditions, the need for support services, and caregiving roles. Its development was grounded in a synthesis of qualitative research findings, from which initial candidate items were drawn. These were subsequently expanded and refined through consultation with diverse stakeholder groups [9, 10]. Both qualitative and quantitative methods were employed to assess face validity and psychometric properties of the measure across six countries (Argentina, Australia, China, Germany, UK, USA) [10, 11]. Two versions of the EQ-HWB are currently available as a self and proxy measure: a comprehensive 25-item profile measure and a more concise short form, the EQ-HWB-9, consisting of nine items.

The number of studies validating the long and short form of the EQ-HWB is steadily increasing [11–15], including work in residential aged care to assess the perspective of staff proxies [8]. However, there is currently no study that captures explicitly the proxy perspectives of informal caregivers of community-dwelling PlwD in Germany. Yet, their viewpoint is of particular importance, as informal caregivers often become the primary source of health-related information when the affected individuals are no longer able to self-report their health and wellbeing state. Therefore, it is crucial to examine the validity of such instruments when used by caregivers, especially in the context of dementia, where frequent day-to-day fluctuations may influence the proxy’s response behaviour.

Health-related measures differ not only in terms of content (e.g. generic vs. specific) [16], but also in the recall period (e.g. today, last week, last 14 days) they assess [17]. There is no established gold standard for the optimal length of the recall period [18]. Instead, it depends on multiple factors, including the purpose and intended use of the measure (e.g. acute effects of an intervention vs. longer-term effects), the health condition with its frequency and fluctuation of symptoms, the accuracy of the instrument and the individual’s ability to recall the requested information correctly and easily [17, 18]. The appropriateness of the recall period’s length may also vary depending on whether the assessment is made by the individual themselves or by a proxy. A previous study on health fluctuations in PlwD found that informal caregivers are more likely to adhere to a “today” recall period when the health of the PlwD is relatively stable [19]. However, a greater amount of day-to-day variations in the caregivers’ perception of the patients’ condition was associated with a higher non-adherence to the today recall period [19], which could indicate the appropriateness of a longer one.

To meaningfully assess the validity of the EQ-HWB-9 in this context, it is essential to investigate how informal caregivers interpret the questionnaire items and how different recall periods reflect the fluctuating nature of dementia, as it may affect the validity and comparability of health-related assessments in such conditions. Therefore, the purpose of this study was to assess the face validity of the EQ-HWB-9 in informal caregivers and to explore how caregivers understand and interpret the EQ-HWB-9 items when using two different recall periods (today vs. 7-days).

## Methods

### Participants

The participants in the study were PlwD and their informal caregivers (dyads), who were recruited in the North of Germany (Mecklenburg-Western Pomerania, Hamburg, Berlin, Brandenburg) between May 2023 and December 2024 through qualified study nurses working with general practitioner practices and memory clinics in ongoing studies with the German Center for Neurodegenerative Diseases (DZNE). Eligibility was determined by study nurses and required either a positive DemTect screening (score ≤ 8) [20] or a formal dementia diagnosis confirmed by a treating physician. Caregivers were eligible and invited to the study if they lived with the PlwD or provided daily care to them.

### Study design

Qualitative interviews were conducted as part of a mixed-methods study that examined the variability and reliability of the EQ-HWB-9 and the influence of health fluctuations in PlwD by using different recall periods. Caregivers and their PlwD were divided randomly into two groups with different recall periods (7 days vs. today). Each participant (caregiver as well as PlwD) completed the EQ-HWB-9 at three time points across 14 days (day 1, day 7, and day 14). The caregivers were instructed to assess the health and wellbeing of PlwD from their own point of view, following the proxy-proxy perspective. This approach captures how the informal caregiver personally perceives the health status of the person they care for. In contrast, the proxy-person perspective asks caregivers to rate how they believe people with dementia would rate their own health, if they were able to do so [21]. Between the three time points, the caregivers were asked to fill-out a daily health fluctuation diary to document day-to-day variations of the PlwD over 14 days. Following the final survey, caregivers participated in in-depth interviews designed to evaluate the appropriateness and usability of the EQ-HWB-9, with a focus on the two different recall periods. The interviews conducted are the basis for the presented analysis.

### Structure of the interview guide

The interviews were structured using a pre-defined interview guide (for the recall period “Today” and the recall period “7-days”) developed by the research team. To minimize participant burden and ensure comprehensibility, especially given the cognitive and emotional demands of the target group, interviews were kept brief and included a mix of open and closed-ended questions. The semi-structured interview guide was organized into four thematic sections: (I) introduction to the interview, (II) perceived changes in the health of PlwD, (III) reflections on the health and wellbeing questionnaire (EQ-HWB-9), and (IV) the recall period. Visual materials, such as a printed version of the EQ-HWB-9 and a list of its dimensions, were used to support participants in expressing their views. The interview guide can be found in Supplementary 2 (Recall period “Today”) and Supplementary 3 (Recall period “7-days”). Two study nurses trained by our research team conducted the interviews at the caregivers’ homes. Regular team meetings were held to discuss challenges encountered during the interviews.

### Procedure of data analysis and definition of face validity

Audio-recorded interviews were transcribed verbatim, and qualitative content analysis according to Mayring [22] was applied using MAXQDA 24 software. All transcripts were read line by line with relevant information coded as it was identified. The coding was done deductively (coding framework based on the interview guide) and inductively (new themes derived from what has been said). The first ten interviews (five on recall period “today” and five on recall period “seven days”) were coded by two researchers (MB, LE). Afterwards, the two researchers met to compare and discuss their codes to refine the structure of the coding framework. The coding framework was then used to guide the coding of the subsequent interviews, which was conducted by one researcher (MB). The second researcher (LE) screened randomly selected interviews (*n* = 11) based on the joint coding framework. Any discrepancies on code application or unclear passages were discussed until consensus was reached. Face validity was assessed focusing on caregivers’ perceptions of the instrument’s acceptability (suitability to capture the health and wellbeing of PlwD, ease of completion, suitability of the measure’s dimensions, and frequency of administration) as well as the perceived appropriateness and adherence of the two recall periods. While frequency of administration is not typically considered part of face validity, it was explored in the study as part of caregivers’ overall appraisal of the EQ-HWB-9’s suitability for use.

### Ethical considerations

Each dyad received detailed oral and written information about the goal, purpose and procedure of the study and gave written informed consent. The study was approved by the Institutional Review Board at the University Medicine Greifswald (BB 030/23) and conducted according to the Declaration of Helsinki.

### Measures

#### EQ-HWB-9

The present study employed the experimental EQ-HWB-9 (2022) version, a short version of the EQ-HWB which consists of nine items with five response options each (frequency or degree of intensity), measuring activities of daily living, mobility, pain, exhaustion, loneliness, concentration, sadness/depression, anxiety, and loss of control [8]. To explore the influence of the recall period on assessments, we applied two different recall frames: one group of caregivers used a “today” recall period, and the second group the original 7-day recall period. The selection of the EQ-HWB-9 was guided by its brevity, aiming to minimize respondent burden for the target population.

#### Sociodemographic data, cognitive status and caregiver burden

Besides sociodemographic variables of the dyads (age, gender, relationship of dyads) we assessed the cognitive status of the PlwD using the Mini-Mental State Examination (MMSE) [23, 24], ranged between 0 and 30, with lower values indicating higher cognitive impairment (30 − 26 mild to no hint for cognitive impairment, 25 − 20 mild, 19 − 10 moderate and ≤ 9 severe). The burden of informal caregivers was assessed using the 7-item Zarit Burden Interview (ZBI), ranged between 0 and 28, where higher scores indicate a more severe caregiver burden [25].

## Results

### Sample description

73 interviews were successfully conducted and included in the analysis; one interview could not be performed. The interviews lasted between 5 and 18 min, with an average of 9 min each. In Table 1 characteristics of the sample are depicted. Informal caregivers had a mean age of 70.6 years, all of whom were providing care for a community-dwelling relative diagnosed with a form of dementia (on average moderately cognitively impaired based on MMSE score), aged on average 77.3 years. The majority of caregivers were female (72.6%), most commonly the spouse or partner (78.1%) of the person being cared for. The ZBI-7, with a mean score of 8.4, indicated mild to moderate caregiver burden. 

**Table 1:** Characteristics of the informal caregivers and their relatives with dementia (PlwD)

Variables	Informal caregivers (*n* = 73)	PlwD (*n* = 73)
Age, mean ± SD	70.6 ± 10.6	77.3 ± 8.0
Gender, female % (n)	72.6 (53)	42.5 (31)
Relationship to the PlwD		
Wife/partner Husband/partner Child Mother Sister Friend	50.7 (37) 27.4 (20) 15.1 (11) 4.1 (3) 1.4 (1) 1.4 (1)	
Caregiver Burden ZBI-7, mean ± SD, range	8.4 ± 5.4, 0–26	
MMSE, mean ± SD, range 0–9 (severe, n, %) 10–19 (moderate, %) 20–25 (slight, n, %) 26–30 (mild to no, n, %)		18.7 ± 7.3, 0–30 9 (12.3) 26 (35.6) 26 (35.6) 12 (16.4)

Note. ZBI-7: Zarit Burden Interview with seven items [25]; MMSE: Mini-Mental State Examination [23, 24]; n: number of observations; SD: Standard Deviation

**Fig. 1:**
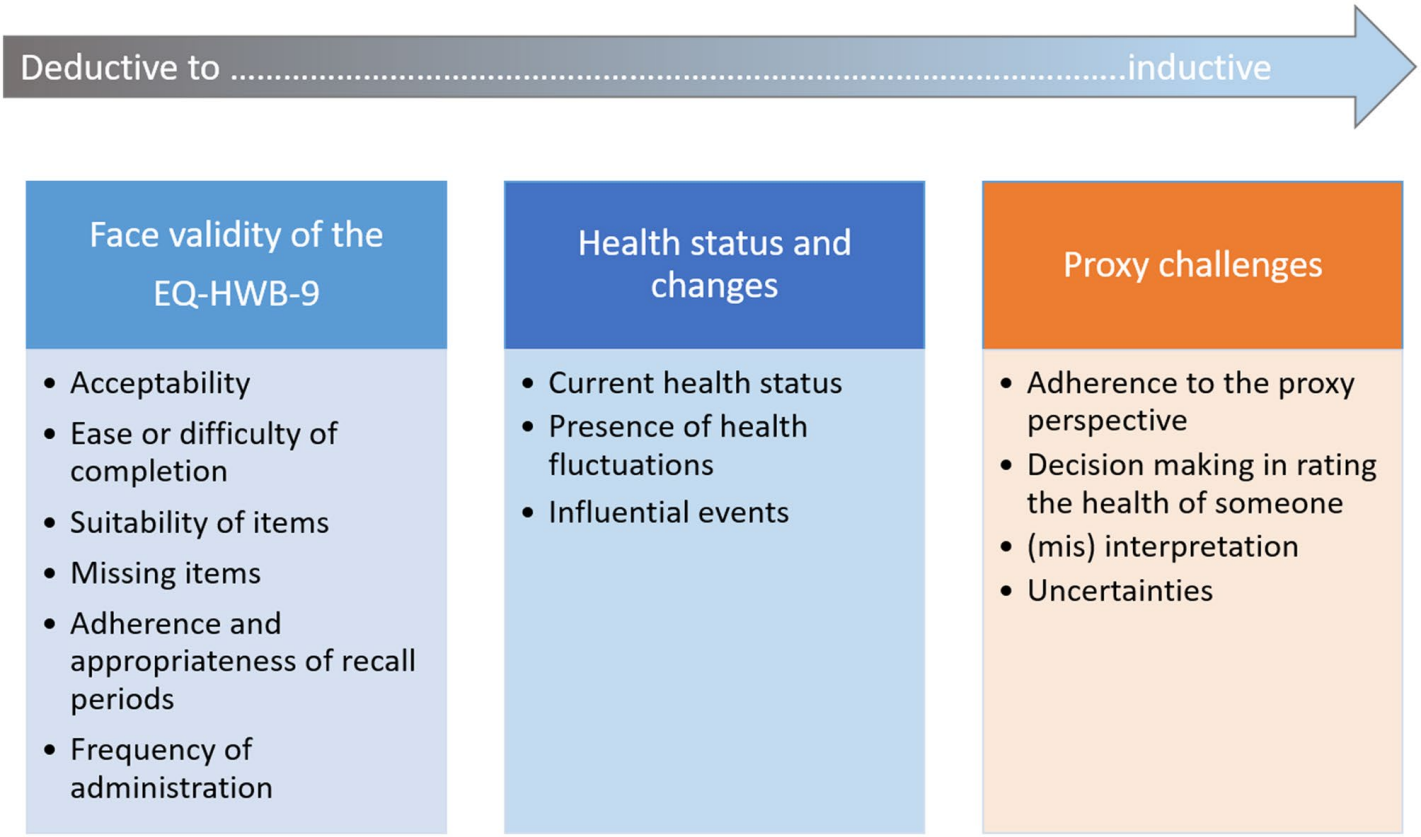
Overview of the extracted categories with sub-themes. Note. The arrow indicates the analytical progression from deductive (interview guide-based) to inductive (data-driven) theme development

As Fig. 1 shows, three themes were found: first, the “face validity of the EQ-HWB-9”; second, “health status and changes”; and third, “proxy challenges”, all with different sub-themes, developed both deductively and inductively. Findings are discussed below according to these main categories.

### Face validity

#### Acceptability

Acceptability was explored by asking caregivers how well they think the EQ-HWB-9 adequately represented their relative’s health and wellbeing. A total of 88% (*n* = 64) of caregivers rated the EQ-HWB-9 questionnaire as acceptable, while 7% (*n* = 5) considered it as moderately and 4% (*n* = 3) as not acceptable. There were no differences between the recall groups. Among those who expressed moderate or negative/no acceptability, common criticisms included the brevity of the questionnaire, the perception that not all relevant health dimensions were covered and that it was too superficial to provide deeper insights into the patient’s health status, as one participant noted:

*They do not know anything specific about him. An outsider cannot truly assess what I’m dealing with here.*

(Participant_44_7-day)

Caregivers also expressed that some questionnaire items are complicated to rate, as they require insight into the internal emotional or physical state of the PlwD – something that is not always directly observable. To make such assessments, they often have to rely on interpreting indirect signs, such as facial expressions, gestures, or changes in behaviour, especially when judging aspects like pain, loneliness, or sadness.

*Whether he’s in physical pain. That’s hard to assess. Sometimes he contorts his face or curls up*,* but it’s difficult to tell.*

(Participant_21_today)

In contrast, those who rated the questionnaire as acceptable emphasized its broad coverage of health dimensions, its clear structure, and the five-point response scale, which they felt allowed for adequate differentiation.

*I’d say that it’s probably a good thing because you have written questions where you can really describe the current condition. So*,* I think it’s quite suitable for that.*

(Participant_1_today)

I found this questionnaire quite good because it was fairly differentiated, in that you actually had five options to respond to.

(Participant_58_7-day)

#### Ease or difficulty of completion

When asked about the difficulty of responding (71 out of 73 answered the question), the majority of informal caregivers (62%, n = 44/71) reported that they found the questionnaire items easy to answer. In contrast, 28% (n = 20/71) described the process as moderately difficult, while 10% (n = 7/71) found it challenging. Caregivers who experienced moderate to high difficulty often cited two main challenges: First, the inherent difficulty of evaluating someone else’s health – underscoring the well-documented complexity of proxy assessments; and second, uncertainty in selecting appropriate response options, particularly when distinctions between terms like “only occasionally” and “sometimes” felt vague or difficult to apply.

Yes, that’s exactly it. How do you judge that? What counts as ‘sometimes’ and what is really just ‘only occasionally’?

(Participant_64_7-day)

Additionally, some caregivers noted that certain items did not accurately reflect the lived reality of the PlwD they were caring for. Their unfamiliarity with standardized assessment formats further contributed to feelings of hesitation and insecurity during the rating process.

#### Suitability of items

**Fig. 2:**
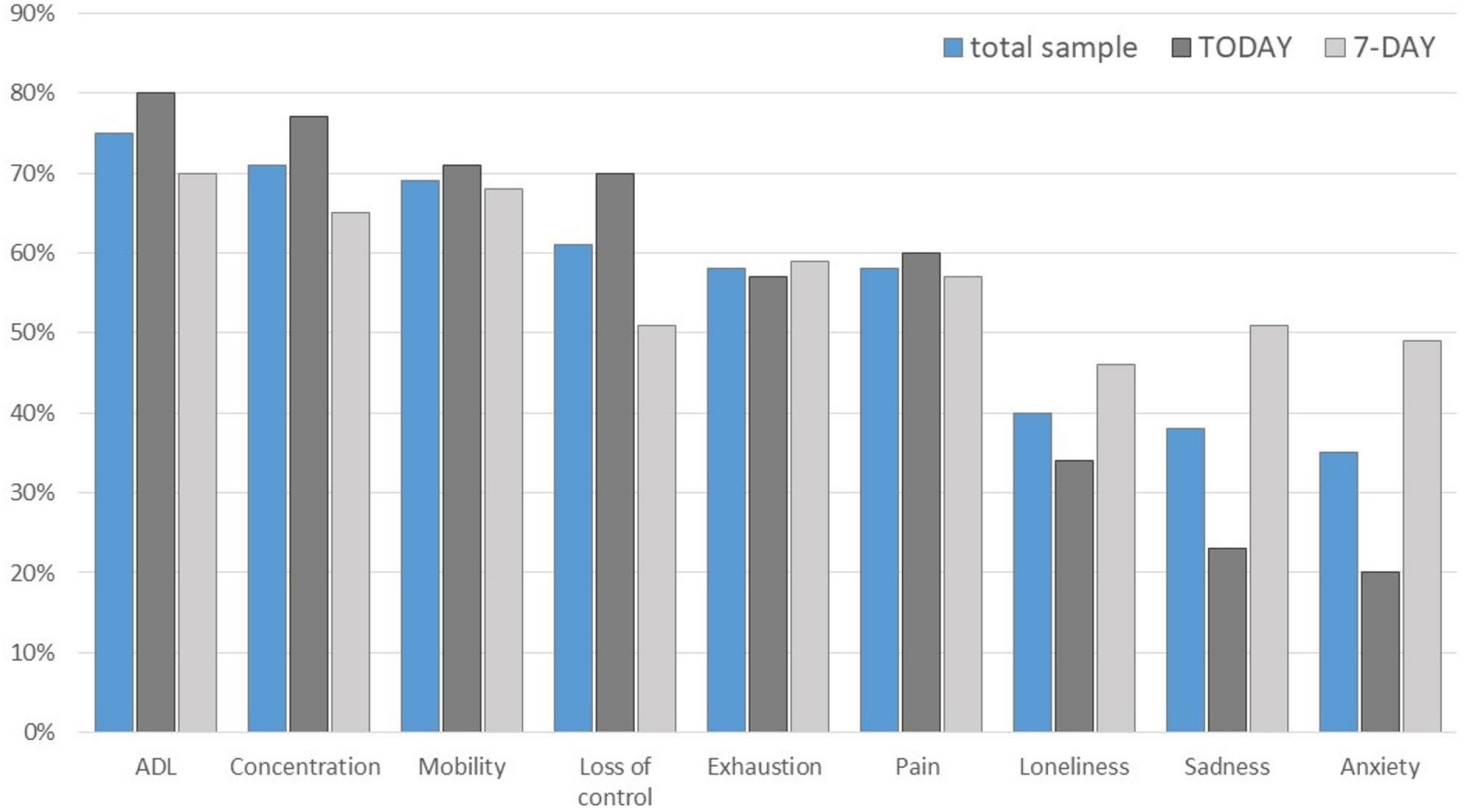
Dimensions of the EQ-HWB-9, perceived suitable to describe the health status (total sample and grouped by recall period). Note. TODAY: Caregivers assigned to the ‘today’ recall period; 7-DAY: Caregivers assigned to the ‘7-day’ recall period. ADL: Activities of daily living. Participants were shown the questionnaire dimensions and asked which they found most suitable to assess their relative’s health and wellbeing. The percentages indicate that the dimension was assessed as suitable

In Fig. 2, the dimensions of the EQ-HWB-9, perceived as suitable to describe the health and wellbeing status, are depicted. Caregivers tended to base their assessment often on more observable aspects such as activities of daily living (75%), concentration (71%), and mobility (69%), over less visible dimensions as loneliness (40%), sadness (38%) or anxiety (35%) to describe the PlwDs’ health status (Fig. 2). The latter were found to require the incorporation of additional, non-observable information, such as PlwD’s mimics (facial expressions), gestures, and behaviours, to provide an accurate assessment.

For example, when it comes to pain—he doesn’t say much, because he speaks very little, and especially not about pain. You have to read it entirely from his facial expressions.

(Participant_22)

Interestingly, caregivers, who were assigned to the 7-day recall period version, differed in their views towards the dimension relevance when compared to the ones assigned to the today recall period, by reporting loss of control as less relevant (51% vs. 70%). Regarding items that were perceived as difficult to answer, caregivers most frequently mentioned the dimensions loneliness (15%, *n* = 11), pain (15%, *n* = 11), and feeling sad or depressed (11%, *n* = 8). Over half of the caregivers (55%, *n* = 40) indicated that no relevant dimensions were missing from the questionnaire. Among those who identified missing aspects, the most frequently mentioned, were sleep, communication, and whether events (e.g., visitors, medical appointments) occurred (Supplementary Table 1).

#### Adherence and appropriateness of recall period

**Fig. 3:**
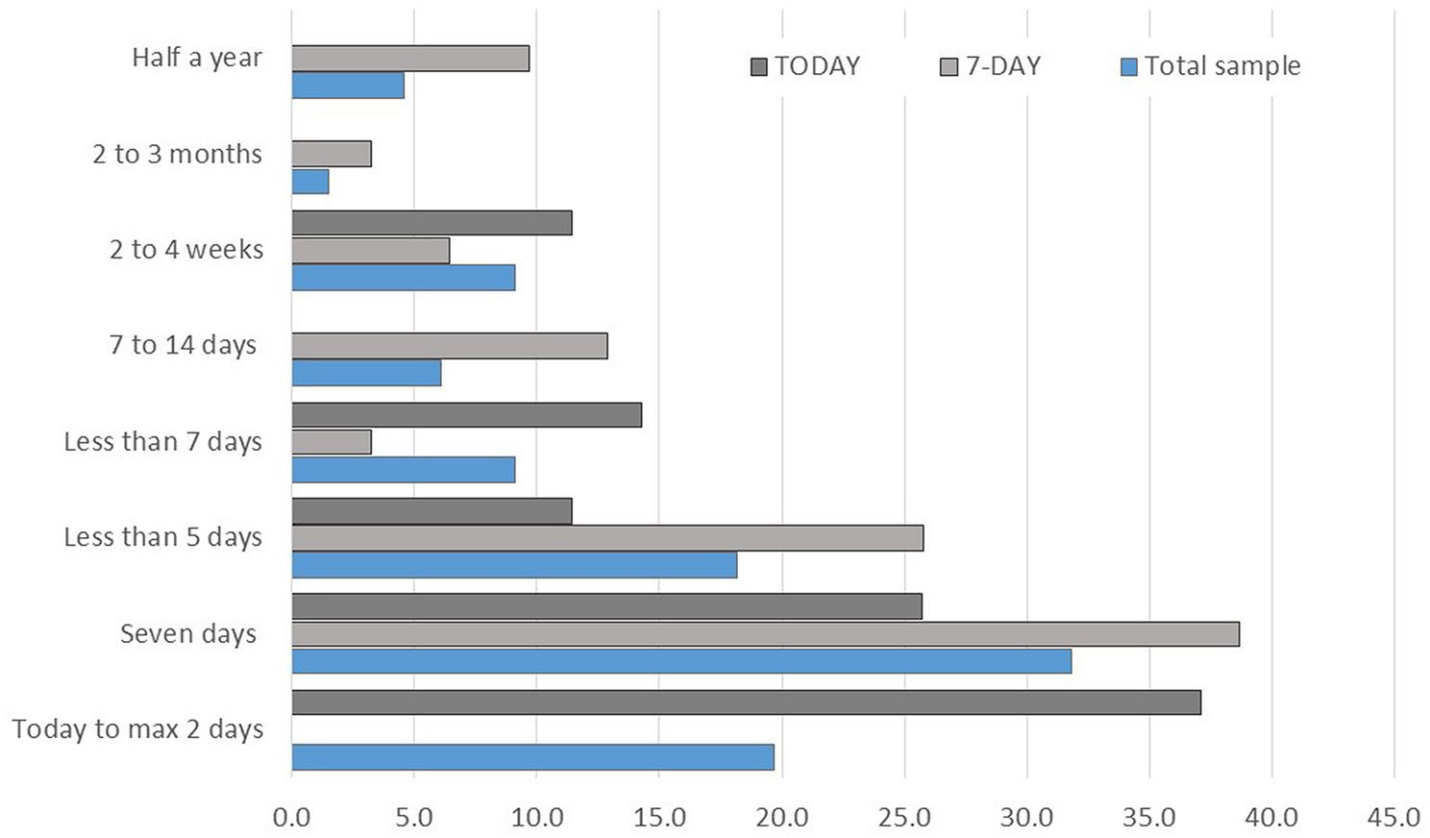
Appropriate recall period based on caregivers’ view, for the total sample and by recall period (%)

Adherence was explored by asking informal caregivers if they adhered to the respective recall period (today or 7-day) when responding to the EQ-HWB-9. Among caregivers in the “Today” group, 86% (n = 32/37) adhered to the instructed recall period. Only one caregiver reported not to adhere, while another generally adhered but occasionally included additional days when assessing the health and wellbeing of the PlwD. Three caregivers reported that they generally adhered to the ‘today’ recall period, although they occasionally considered one or more additional days when responding to the EQ-HWB-9. In the “seven-day” recall period group, 76% (*n* = 26/34, two caregivers did not give an answer to the question) strictly followed the recall period, while four participants reported to adhere “mostly”, and another four did not adhere to the instructed period. When asked why they adhered, most caregivers – regardless of group – said they aimed to follow the questionnaire’s explicit instructions. Caregivers who reported non-adherence explained that a longer or shorter recall period felt more appropriate to capture accurately the PlwD’s health and wellbeing. When asked about their preferred recall period (Fig. 3), the largest proportion of caregivers overall (31.8%), as well as within the 7-day recall period group (38.7%) favoured a seven-day period. This was followed by a preference for “today” or up to two days (19.7% total sample; 37.1% in the today recall period group), with fewer caregivers selecting shorter than five days. Interestingly, caregivers generally preferred the recall period to which they had been assigned, suggesting that recall framing may influence perceptions of appropriateness. 

#### Frequency of administration

Frequency of administration was assessed by asking caregivers how often the EQ-HWB-9 should be administered to get a reliable understanding of their relative’s health and wellbeing. In total, 59 caregivers answered this question. 34% of caregivers (*n* = 20/59) preferred weekly administration of the EQ-HWB-9, while others suggested administering it twice a year (12%) or quarterly (10%). Less common preferences included shorter or longer intervals, such as biweekly, monthly, or annual administration, indicating heterogeneity in perceived feasibility and appropriateness of the assessment frequency.

No, once a week is enough. I think that’s good for both (caregiver and PlwD).

(Participant_10_7-day)

### Health status and changes

During the study period, 44% stated that the health of their relative had changed due to various disease-related reasons, such as swallowing problems, mobility issues, epileptic seizures, or sickness caused by the flu. Most caregivers rated such behaviour as typical (85%) and described situations with both good and bad days, which is common in dementia.

Because it varies a lot. Sometimes she’s in a good mood, sometimes not. Sometimes she’s alert, and other times she’s withdrawn. But that’s been the case for quite a while.

(Participant_45)

Furthermore, the caregivers reported that special events influenced the PlwDs’ behaviour both positively and negatively. Most often, visits from family members or friends, as well as activities (e.g., outdoor or cultural events), had a positive impact on the PlwD’s mood. Negative impacting events included when the caregiver went out, fell in front of strangers, or had some medical or therapeutic appointments (e.g., general practitioner).

### Proxy challenges

#### Adherence to the proxy-proxy perspective

Overall, adherence to the instructed proxy-proxy perspective did not pose a significant issue. Caregivers generally understood the task well and tried their best to follow the instructions to assess the health and wellbeing of the PlwD from their own perspective. However, caregivers described the tension between strictly adhering to the proxy-proxy perspective and providing responses that reflected the health status of PlwD. Caregivers reported that some items were relatively easy to answer. In contrast, others, particularly those concerning pain, sadness/depression or anxiety, were extremely challenging, especially in cases where the PlwD was no longer able to communicate effectively. Some participants expressed uncertainty when selecting response options, struggling to decide which answer best captured the situation from their own perspective, including the knowledge that their rating could differ significantly from the PlwDs’ self-rating. This uncertainty highlights the inherent challenges of proxy reporting, particularly when caregivers are asked to assess the health of another person. The following two statements illustrate this challenge clearly:

If I rate her too poorly, it reflects badly on my mother. But if I rate her too positively, then it’s not entirely accurate either.

(Participant_55)

The questions that are meant for the other person are very difficult for me to answer. I can only judge them from my own point of view. So, it might not actually reflect the patient accurately.

(Participant_60)

Furthermore, the quality of the relationship between the PlwD and the caregiver proved to be a key factor in how challenging caregivers found it to assess someone else’s health and wellbeing status.

Well, since we have a very close relationship, I had no difficulty answering at all. No problems. It was relatively easy.

(Participant_25)

## Discussion

This study aimed to explore the face validity of the EQ-HWB-9 as a proxy measure for assessing the health and wellbeing status of community-dwelling PlwD as perceived by their informal caregivers. In this analysis, we identified that the EQ-HWB-9 is generally suitable as a proxy measure. Although not a primary focus of this study, the role as a proxy also poses challenges with the instrument’s recall period and content, particularly when caregivers must interpret non-verbal signs.

### Acceptability, appropriateness and adherence

The participating caregivers were typically female and older adults (mean age ~ 70 years), which reflects the broader demographic profile of informal caregivers in dementia care [26, 27]. The overall acceptability of the EQ-HWB-9 was high; caregivers generally found the instrument easy to understand and complete. However, some caregivers criticized the lack of dementia-specific content, noting that disease-related aspects, such as sleep and communication, were not adequately addressed. This critique, while valid, must be contextualized: the EQ-HWB-9 is designed as a generic measure and not intended to assess condition-specific symptoms [28]. Generic instruments are designed for use in the general population and across various disease groups, enabling comparisons of the impact of different health conditions and facilitating their application in health economic evaluations [29]. In contrast, disease-specific measures are tailored to capture quality of life aspects particularly relevant to a specific condition, generally offering greater sensitivity to clinically meaningful changes [29]. Although previous studies analyzed the suitability of different generic measures for use in dementia populations [30–32], evidence to establish the validity of the EQ-HWB-9 to disease-specific instruments in this context is still needed. Additionally, it has to be noted, that the long version EQ-HWB-25 captures some of the aspects that caregivers found missing in the short form (i.e., sleep and communication) [33], suggesting that the item reduction process to create the short version may have excluded content of particular relevance for this population.

Furthermore, some of the caregivers reported that the instrument provides only a superficial view of the PlwD’s health status. As a measure of subjective health and wellbeing, the aim of the EQ-HWB-9 is to provide a broad overview, not an in-depth clinical/disease assessment. The reported criticism by caregivers could also be explained by a lack of knowledge about the purpose of such instruments, as stated in some interviews, and unfamiliarity with these surveys. This should not be interpreted as indicating any deficiency on the part of the caregivers. It must be acknowledged that informal caregivers typically lack professional healthcare training, which may limit their ability to grasp the purpose of such assessments fully. Instead, the instrument’s instructions should be clearly formulated, and the study personnel should thoroughly explain the study’s objectives and the intended use of the measure. A recently published study highlights the importance of such instructions in enabling proxies to report the health of others more accurately [34].

Another factor that complicated the completion of the EQ-HWB-9 was the requirement to adhere to a specific recall period. Norquist et al. [17] extensively discussed the selection of appropriate recall periods for patient-reported outcomes (PRO), emphasizing that the chosen timeframe should account for the respondent burden and the individual’s ability to accurately and easily recall the requested information. This becomes particularly challenging in conditions with day-to-day variations, such as dementia, where both excessively short and long recall periods may lead to a biased representation of the respective health status. Despite these considerations, there is currently a lack of research specifically addressing the suitability of recall periods in PROs from the perspective of informal caregivers of PlwD. Our findings contribute to this gap by providing initial insights into how caregivers experience and accept different recall periods. In our data, the seven-day recall period was associated with lower adherence compared to the ‘Today’ group. Caregivers who reported non-adherence to the recall instructions mainly tended to prefer shorter recall periods. Notably, the majority of caregivers expressed that recall periods that are longer than seven days would be inappropriate, further emphasizing the need to tailor recall periods in this population carefully. Furthermore, the length of the recall period appears to have an effect on less observable dimensions (e.g. concentration) which should be considered in questionnaire design and recall period selection.

### Proxy challenges and response uncertainty

The difficulty of evaluating someone else’s health and wellbeing emerged as a critical issue. Caregivers expressed uncertainty when selecting response options, particularly in emotional domains (e.g., anxiety, sadness/ depression) and physical aspects such as pain, limiting an accurate health and wellbeing rating of the PlwD. Caregivers have to interpret the gestures, facial expressions, and behaviors of their PlwD to make an adequate assessment. Such interpretations could lead to uncertainty among some caregivers and may result to biased responses. An indicator of response uncertainty can be observed in the response pattern of the items. Previous studies suggest that informal caregivers often display a central tendency bias, preferring mid-range response options and avoiding the extreme ends of a scale, possibly reflecting uncertainty [8, 35]. Such uncertainties may undermine the reliable application of the proxy-proxy perspective, potentially leading caregivers to deviate from this perspective when rating health unconsciously. However, the specific direction of such deviations remains unclear, as informal caregivers may rely on their own judgment or on assumptions about how the PlwD would evaluate their own health and wellbeing. In the interviews, it became evident that assessing the health of a close family member may further complicate adherence to the proxy-proxy perspective due to emotional and relationship-related aspects. Additionally, shift effects may bias proxy reports, meaning that caregivers’ own emotional or physical well-being could inadvertently affect how they rate the health status of the care recipient [36, 37]. This effect is particularly relevant for informal caregivers, who often experience high levels of stress and burden. In our sample, the measured caregiver burden was on a mild to moderate level, nevertheless we could not exclude its influence.

### Strengths and limitations

Several limitations should be considered when interpreting the findings of this study. First, the interviews were conducted by study nurses rather than researchers. While this may have encouraged caregivers to speak more openly due to the perceived familiarity and reduced formality, it could have reduced the depth of questioning and the exploration of more complicated issues. To address this, the study nurses received targeted training in qualitative interviewing techniques, and the interview guide was discussed extensively to minimize the risk of misinterpretation. Furthermore, regular meetings of the study team (researchers and study nurses) were held throughout the data collection phase to discuss any issues that arose during the interviews, ensuring continuous reflection and improvement of the interview process. Second, the interviews were part of a larger mixed methods study, which may have influenced the caregivers’ responses. Specifically, caregivers often preferred the recall period to which they had been assigned, suggesting that their perception of appropriateness may have been shaped by the study’s framing. It is possible that a standalone qualitative study, without the surrounding quantitative structure, could have yielded different insights. Third, this study examined a proxy-proxy rather than a proxy-person perspective. These two perspectives may differ substantially in how caregivers rate the health of their relative with dementia. Previous studies have shown [21, 38, 39], that discrepancies between self- and proxy rated health tend to be smaller when using a proxy-person perspective, highlighting the need for future research to systematically compare both proxy perspectives. Fourth, the retrospective interview design may have introduced recall bias. Adherence to the instructed recall period was based on self-reports and could not be verified, meaning that some informal caregivers may not have fully attended to the recall instructions. Future studies could use eye-tracking methods to better assess this aspect, as shown by Milte et al. [40]. The study included patients with various types of dementia. Since different dementia disorders are associated with distinct patterns of health fluctuations and symptom progression, this heterogeneity may have introduced variability in the challenges reported by caregivers. However, as the specific type of dementia was not assessed, we are unable to determine whether or how the dementia type influenced caregivers’ response patterns. A further limitation is that we did not explore whether cognitive impairment or disease severity influenced caregivers’ perceptions. Finally, while the use of two raters for a subset of interviews improved consistency in coding, the remaining interviews were mainly screened by a single researcher. Including two raters for all interviews in future studies could further enhance methodological rigour and reduce subjective bias.

## Conclusion

This study provides initial evidence that the EQ-HWB-9 is broadly acceptable and feasible as a proxy measure for assessing the health and wellbeing status of PlwD from the perspective of informal caregivers. However, challenges remain regarding the suitability of the recall period, the generic content of the measure, and the inherent difficulty of proxy reporting, particularly when caregivers must interpret non-verbal signs. These challenges may contribute to response uncertainty and potential bias, influenced by the emotional closeness to the care recipient and the caregiver’s well-being. To support accurate proxy reporting, future research should further investigate optimal recall periods for proxy assessments, consider the role of caregiver burden, and ensure that clear instructions and thorough guidance accompany proxy measures.

## Supplementary Information

Below is the link to the electronic supplementary material.

Supplementary Material 1

Supplementary Material 2

Supplementary Material 3

## Data Availability

Data is available on request.
