# Supplementary material for: Examining the face validity of the EQ-HWB-9 in dementia: caregiver interpretation across “Today” and “7-Day” recall periods
Source: Health Qual Life Outcomes. 2026 Jan 28;24:22. doi: 10.1186/s12955-026-02478-z (PMC12924600; doi:10.1186/s12955-026-02478-z)
Supplement: Supplementary file 1 — Supplementary Material 1 [file 12955_2026_2478_MOESM1_ESM.docx]

**Examining the face validity of the EQ-HWB-9 in dementia: caregiver interpretation across "Today" and "7-Day" recall periods**

Maresa Buchholz^1^, Lidia Engel^2^, Feng Xie^3, 4^, Bernhard Michalowsky^1, 3^

^1^Patient-reported Outcomes & Health Economics Research German Center for Neurodegenerative Diseases (DZNE), site Rostock/ Greifswald, Ellernholzstrasse 1-2, Greifswald D-17487, Germany

^2^Monash University Health Economics Group, School of Public Health and Preventive Medicine, Monash University, Melbourne, 553 St Kilda Rd, Melbourne VIC 3004, Australia

^3^ Department of Health Research Methods, Evidence and Impact, McMaster University, Hamilton, Ontario, Canada

^4^ Centre for Health Economics and Policy Analysis, McMaster University, Hamilton, Ontario, Canada

**Contact information for corresponding author**

Dr. Maresa Buchholz

Patient-reported Outcomes & Health Economics Research, German Center for Neurodegenerative Diseases (DZNE), Site Rostock/ Greifswald

Ellernholzstraße 1-2, 17489 Greifswald, Germany

maresa.buchholz@dzne.de

Phone: +49 (0)3834 86 85 32

ORCID: 0000-0002-4486-8632

**Supplementary Table 1.** Frequency of reported missing items

| **Missing items** | **%** |
| --- | --- |
| nothing | 53.33 |
| do not know | 5.33 |
| sleep | 5.33 |
| communication | 4.00 |
| inquire more deeply | 4.00 |
| events | 4.00 |
| wellbeing of the one who takes care | 2.67 |
| eating behaviour | 2.67 |
| forgetfullness | 1.33 |
| lack of motivation | 1.33 |
| question about overall health | 1.33 |
| Where can I get help from others? | 1.33 |
| medication needs | 1.33 |
| how can I help as a relative | 1.33 |
| Emotions | 1.33 |
| Independency | 1.33 |
| social contacts | 1.33 |
| wellbeing | 1.33 |
| Successful experiences | 1.33 |
| behaviour/aggressiveness | 1.33 |
| joint questionnaire | 1.33 |
| what makes fun/ pleasure | 1.33 |
| Total | 100.00 |
